# Mutations of *CREBBP* and *SOCS1* are independent prognostic factors in diffuse large B cell lymphoma: mutational analysis of the SAKK 38/07 prospective clinical trial cohort

**DOI:** 10.1186/s13045-017-0438-7

**Published:** 2017-03-17

**Authors:** Darius Juskevicius, David Jucker, Dirk Klingbiel, Christoph Mamot, Stephan Dirnhofer, Alexandar Tzankov

**Affiliations:** 1 Institute of Pathology, University of Basel and University Hospital Basel, Schoenbeinstrasse 40, CH-4031 Basel, Switzerland; 2 Swiss Group for Clinical Cancer Research (SAKK), Effingerstrasse 40, CH-3008 Bern, Switzerland; 3 Department of Biomedicine, University Hospital Basel, Hebelstrasse 20, CH-4031 Basel, Switzerland; 4 Center of Oncology, Hematology and Transfusion Medicine, Cantonal Hospital Aarau, Tellstrasse 25, CH-5000 Aarau, Switzerland

**Keywords:** DLBCL, Prognostic markers, Targeted high-throughput sequencing, NGS, Lymphoma, *CREBBP*, *SOCS1*, *EP300*

## Abstract

**Background/purpose:**

Recently, the mutational background of diffuse large B cell lymphoma (DLBCL) has been revealed, identifying specific genetic events that drive lymphomagenesis. However, the prognostic value of these mutations remains to be determined. Prognostic biomarkers in DLBCL are urgently needed, since the current clinical parameter-based factors (e.g., International Prognostic Index (IPI)) are insufficient, particularly in identifying patients with poor prognosis who might benefit from alternative treatments.

**Methods:**

We investigated the prognostic value of somatic mutations in DLBCL in a clinical trial (NCT00544219) patient cohort homogenously treated with six cycles of rituximab, cyclophosphamide, hydroxydaunorubicin, vincristine, and prednisone (R-CHOP), followed by two cycles of R (R-CHOP-14). The primary endpoint was event-free survival (EFS) at 2 years. Secondary endpoints included progression-free survival (PFS) and overall survival (OS). Targeted high-throughput sequencing (HTS) of tumor genomic DNA was performed on all exons or hotspots of 68 genes frequently mutated in B cell lymphomas. Mutational data was correlated with the endpoints to identify prognostic associations.

**Results:**

Targeted HTS detected somatic mutations in 71/76 (93%) of investigated cases. The most frequently mutated genes were *KMT2D*, *SOCS1*, *GNA13*, and *B2M*. Survival analysis revealed that *CREBBP-* and *EP300*-mutated cases had significantly worse OS, PFS, and EFS. In addition, *ATM* mutations predicted worse outcomes for all three clinical endpoints in germinal center B cell-like DLBCL. In contrast, *SOCS1* mutations were associated with better PFS. On multivariable analysis taken into account IPI and failure to achieve complete remission, *CREBBP* and *EP300* mutations remained significant to predict worse OS, PFS, and EFS.

**Conclusion:**

Targeted mutation analysis of a uniformly treated prospective clinical trial DLBCL cohort identifies tumor-based genetic prognostic markers that could be useful in the clinical management of such patients.

**Trial registration:**

ClinicalTrials.gov NCT00544219

**Electronic supplementary material:**

The online version of this article (doi:10.1186/s13045-017-0438-7) contains supplementary material, which is available to authorized users.

## Introduction

Diffuse large B cell lymphoma (DLBCL) is a heterogeneous and aggressive lymphoid neoplasm, the treatment of which has significantly improved in the last decade with addition of the anti-CD20 monoclonal antibody rituximab (R) to the chemotherapy regimen consisting of cyclophosphamide, hydroxydaunorubicin, vincristine, and prednisone (R-CHOP) [1]. Since then, R-CHOP has become a standard treatment for the vast majority of primary DLBCL cases with cure rates at about 60%. The remaining 40% of DLBCL either relapses after a period of remission or are refractory to the applied first-line therapy. Patients in this group are treated with aggressive salvage regimens supported by autologous stem cell transplantation (ASCT) [2], but success rates are modest, particularly in primary refractory DLBCL [3, 4]. Therefore, it is important to identify high-risk patients before administration of first-line therapy so that the potentially more aggressive tumors can be treated with alternative regimens [5].

Risk stratification of DLBCL has relied for more than 20 years on the International Prognostic Index (IPI), which is based on evaluation of multiple clinical parameters [6]. Following the addition of rituximab, IPI was revised (R-IPI) [7] and, most recently, further enhanced (NCCN-IPI), to better identify high-risk patients [8]. According to NCCN-IPI, the high-risk group has 5-year overall survival (OS) probability of 33% compared to 54% predicted by IPI, although this remains to be confirmed by the datasets of prospective trials.

In addition to patient-based factors, tumor-based prognostic markers have been proposed. It is known that ~10% of DLBCL cases have *MYC* rearrangements that are strongly associated with worse outcomes, especially if linked to MYC protein overexpression [9]. It has also been shown that the activated B cell (ABC) cell-of-origin (COO) DLBCL subtype has a worse outcome compared to the germinal center B cell (GCB) subtype [10]. Because original classification based on gene expression profiling on a transcriptome level proved to be technically too challenging for implementation in clinical practice, surrogate immunohistochemistry-based algorithms were developed [11]. However, their utility was limited by suboptimal concordance to the gene expression-based gold standard. Recently, a Lymph2Cx assay, which can measure expression of 20 genes and can be also applied on formalin-fixed paraffin-embedded (FFPE) material, was proposed for reproducible classification of DLBCL into COO subgroups [12]. It remains to be seen if this new technology will be widely accepted in lymphoma centers worldwide. In addition to COO classification, immunohistochemical (IHC) studies have identified multiple protein markers, such as CD5, Ki-67, FOXP1, HLA-I, p21, and CD40, that prospectively showed prognostic value for R-CHOP-treated DLBCL [13–17].

During the last decade, substantial progress has been made toward understanding the genetic basis of DLBCL [18–20]. Shared and COO subtype-specific DNA lesions have been identified, converging into several most frequently dysregulated cellular pathways [21]. Based on these findings, a handful of molecular prognostic markers have been identified, among which, *TP53*, *FOXP1*, and *MYD88* mutations and *CDKN2A* deletions were associated with inferior outcomes in R-CHOP-treated DLBCL [22–24]. The prognostic role of such molecular markers is likely to increase in the near future as high-throughput sequencing (HTS) enters routine practice in many institutions. However, more studies, particularly prospective analyses, are required to validate the existing molecular prognostic markers and to discover new ones that would add power to the existing prognostication algorithms.

In this study, we employed targeted HTS to identify somatic mutations in tumors of a well-documented prospective clinical cohort consisting of uniformly treated primary DLBCL patients. By correlating gene mutation status to the robust survival data, we aimed to discover new, and validate known, prognostic markers in DLBCL.

## Methods

### Study cohort

The clinical trial SAKK 38/07 (NCT00544219), active between 2007 and 2010, included 138 eligible patients with primary untreated DLBCL to prospectively determine the prognostic value of interim PET/CT scans by standardized treatment and evaluation criteria. The main clinical results have been published previously [25].

Tissue specimens of patients who consented for additional translational research were used for a subsequent study investigating the prognostic value of phenotypic and genotypic profiles by IHC and fluorescence in situ hybridization (FISH), the results of which have been recently published in this journal [13].

For the current study, FFPE tissues of 84 primary untreated de novo DLBCL patients with adequate amounts of remaining material were selected. Tumor content was determined by morphological evaluation and was at least 50% in all samples.

Updated clinical data (last follow-up on 31 January 2017) were used for evaluation of the prognostic role of genetic mutations. All patients in the study cohort were uniformly treated with six cycles of R-CHOP, followed by two cycles of R (R-CHOP-14). The primary endpoint was event-free survival (EFS) at 2 years (for definition, see the “Statistical analysis” section), and the secondary endpoints were progression-free survival (PFS) and OS at 2 and 5 years as well as objective responses according to international criteria [26].

### DNA extraction and quantification

Genomic DNA was extracted with the GeneRead DNA FFPE kit (Qiagen, Nussloch, Germany) following manufacturers’ recommendations with minor modifications. Briefly, one to three 10–25 μm thick tissue sections were deparaffinized by several xylene washes, rehydrated and digested with proteinase K overnight at 56 °C in a shaking heat block. Following digestion, the samples were incubated for 1 h at 90 °C to reverse fixation-induced DNA crosslinks and inactivate proteinase K. Thereafter, uracil-*N*-glycosylase (UNG) enzyme was added to remove artificially (formalin) induced uracils and reduce the number of false-positive C > T transitions. After incubation at 37 °C for 1 h, the samples were loaded into a DNA purification column and were washed and eluted in 40 μl of nuclease-free water. DNA yields were quantified with the Qubit High sensitivity DNA assay (Life Technologies, Eugene, OR, USA).

### Targeted HTS sequencing variant calling and filtering

A target enrichment panel was designed to cover mutational hotspots or all exons of genes most frequently mutated in B cell lymphoid neoplasms according to the COSMIC database (release v70) and manual review of the literature [27]. Sequencing libraries were constructed exactly as described previously [27]. One microliter of the prepared library was used for the Bioanalyzer High Sensitivity DNA assay (Agilent, USA) to confirm expected DNA fragment length distribution. Quantification was performed with the Ion Library Quantitation kit (Thermo Fisher Scientific, Carlsbad, CA, USA) following the original protocol. Libraries were diluted to 40 pM, loaded to the Ion540 sequencing chips by automated IonChef instrument, and sequenced with the Ion Torrent S5 XL machine (Thermo Fisher Scientific, Carlsbad, CA, USA). The depth of coverage, coverage uniformity, and number of variants called per sample are summarized in Additional file 1: Table S1. Mutation identification was performed by the Variant caller plug-in v5.0 of the Torrent Suite (Thermo Fisher Scientific) using default low stringency parameters for somatic mutation calling. Mutations were annotated using the Ion Reporter variant annotation workflow v5.0 and dbNSFP v3.0 database [28]. MetaLR rank score was used to predict the functional impact of non-synonymous point mutations to the encoded protein [29]. After annotation, the variants were subjected to additional, more stringent, and quality- and relevance-based filtering by criteria as detailed in Table 1. All variants with variant allelic frequency >5% were used in downstream analysis.

**Table 1:** Criteria used for mutation filtering (variant inclusion)

Criterion name	Threshold value
General quality	
Phred-based quality	>50
Strand bias	≤0.75
Number of reads supporting called variant	≥10
Functional relevance	
Variant allelic frequency	≥5%
Localization	Exonic and splice site
Variant effect	Non-synonymous
SNP exclusion	
Variant allelic frequency	<95%
Database annotation and alternative allelic frequency (1000 genomes project, European descendent samples)	Not listed in dbSNP v138 or listed, but MAF ≤0.01%
Variants in detected in the control cohort of 23 non-tumoral samples from lymphoma patients	Not overlapping

Finally, aligned BAM files were manually inspected at sites of all remaining variants to exclude false-positive mutations or other artifacts introduced during library preparation [30].

### Statistical analysis

All statistical analyses were performed using the Statistical Package of Social Sciences (IBM SPSS version 22.0, Chicago, IL, USA) for Windows. EFS was calculated from registration to progressive disease or relapse, death of any cause, and initiation of any non-protocol anti-cancer treatment because of lymphoma symptoms or need of concomitant radiotherapy. PFS was calculated from registration to progressive disease or relapse, and death of any cause. OS was calculated from registration to death. Patients not experiencing an event were censored at last follow-up. The survival probabilities were determined using the Kaplan–Meier method, and groups were compared using the log-rank test. Factors of prognostic significance in univariable models underwent multivariable analysis using the Cox proportional hazards model. For other endpoints, differences between groups were tested either with *t* test, Wilcoxon rank-sum test, or Fisher’s exact test, as appropriate. In all tests, *p* values are two-sided, considered significant if <0.05, and not corrected for multiple testing. For survival analysis within the COO subgroups, *p* values were corrected for multiple testing and were considered significant if <0.017.

## Results

### Patients and clinico–pathologic characteristics

In total, 84 patient samples were included. Four samples yielded insufficient DNA for HTS library preparation, and another four samples failed quality control after sequencing (low coverage uniformity, low percentage of reads mapped to target), leaving 76 cases for further study. Patient characteristics are presented in Table 2. Survival data was available for all 76 patients. The 2-year OS, PFS, and EFS of the entire cohort were 91% (95% CI 84 to 98%), 78% (95% CI 68 to 88%), and 63% (95% CI 53 to 75%), respectively. There was no significant difference in any of the survival endpoints between the two different immunohistochemically determined COO subtypes. IHC and FISH data on all samples were collected as part of the previous translational study focusing on the prognostic value of genotypic and phenotypic characteristics [13]. “Double-hit score,” representing IHC-based evaluation of tumor cell expression of BCL2 and MYC (Table 2), showed that 10/76 (13%) cases were positive for both MYC and BCL2. None of the investigated cases had chromosomal translocations affecting both *MYC* and *BCL2* genes. Thirty of 76 (39%) and 1/76(1%) of cases were positive for FOXP1 and CD5, respectively, both associated with worse DLBCL outcomes in our previous study [13]. All investigated cases were Epstein–Barr virus (EBV)-negative as determined by in situ hybridization for EBV early RNA (EBER).

**Table 2:** Patient characteristics

Age, median (range)		59 (18–81)
Gender, *N* (%)	F	34 (45)
	M	42 (55)
Stage, *N* (%)	I	7 (9)
	II	24 (31)
	III	22 (30)
	IV	23 (30)
IPI, *N* (%)	0–1	35 (46)
	2	19 (25)
	3	11 (14)
	4–5	11 (14)
Treatment	R-CHOP-14	76
Treatment response according to international criteria [26], *N* (%)	CR	63 (83)
	PR	12 (16)
	SD	1 (1)
	PD	0 (0)
Survival, median (IQR)	PFS	55.05(56.7)
	EFS	55.05 (56.7)
	OS	61.9 (9.65)
Cell-of-origin (Tally) [11], *N* (%)	non-GCB	44 (58)
	GCB	32 (42)
Double-hit score, *N* (%)	0	35 (46)
	1	31 (41)
	2	10 (13)
Translocations *N* (%)	*MYC*	5 (9)
	*BCL2*	6 (11)

### Mutational data

**Fig. 1:**
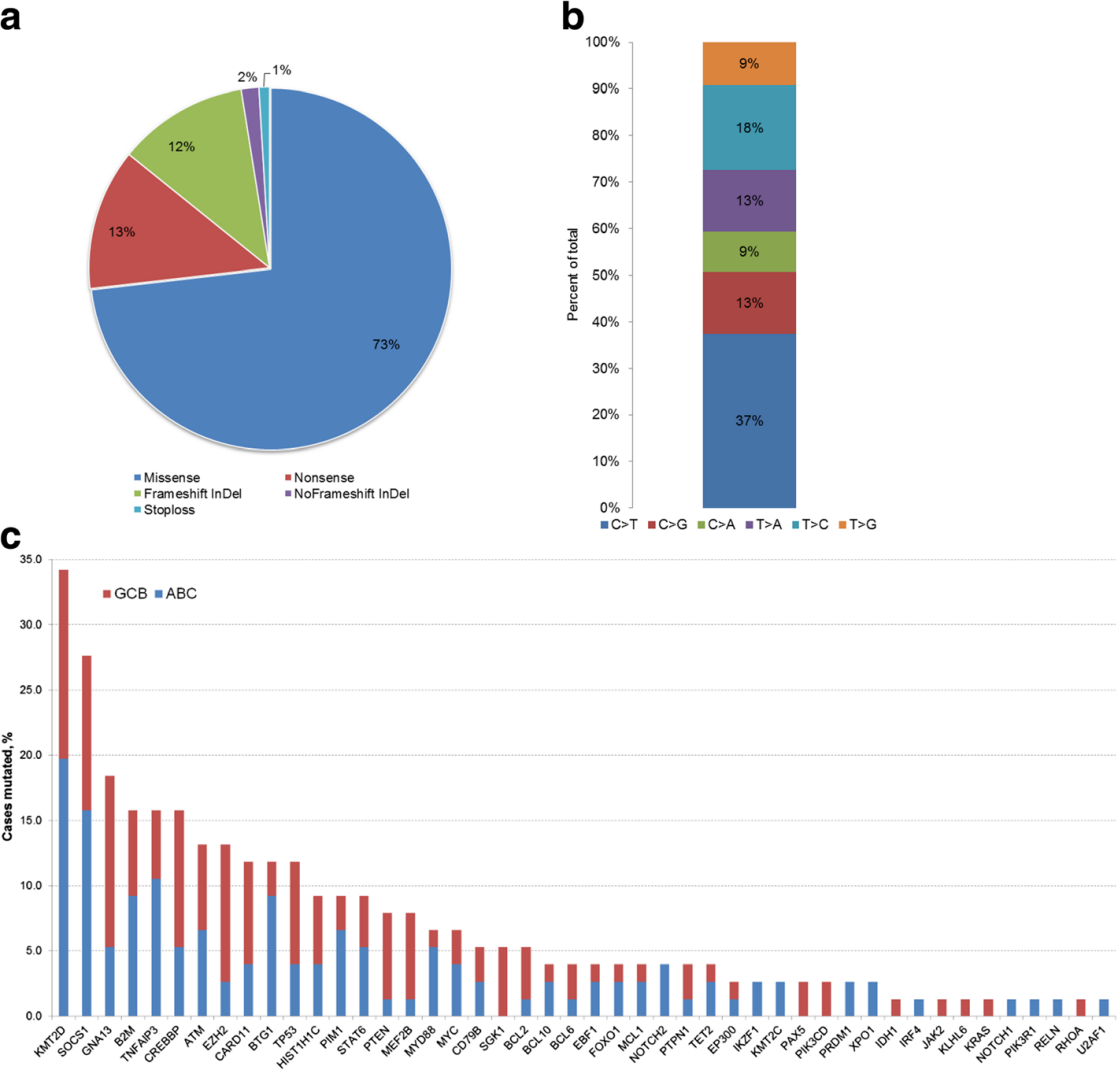
Overview of detected mutations. **a** Classification of the detected mutations according to their type. **b** Frequency of different nucleotide substitutions among all types of point mutations (*n* = 270). **c** Frequency of gene mutations in respect to cell-of-origin classification

In total, 317 somatic alterations were detected. Missense mutations were the most frequent, 232/317 (73%), followed by 40 (13%) nonsense mutations and 37 (12%) frameshift insertions/deletions (Fig. 1a–b and Additional file 1: Table S2). Sixty-eight percent (46/68) of the investigated genes were mutated at least once, and at least one somatic mutation was identified in 71/76 (93%) of investigated tumor samples. The most frequently mutated gene was *KMT2D* (34% of cases), followed by cytokine signaling 1 (*SOCS1*) (28%), *GNA13* (17%), and *B2M* (15%) (Fig. 1c). *EZH2* (*p* = 0.0176) along with *GNA13* (*p* = 0.0181) and *SGK1* (*p* = 0.028) were more frequently mutated in GCB–DLBCL. On average, GCB–DLBCL cases had more mutated genes than ABC-DLBCL cases (mean 3.9 vs 2.8, *p* = 0.018). However, there was no significant difference between COO subtypes considering the cumulative number of mutations per case (some genes had multiple mutations in the same tumor).

**Fig. 2:**
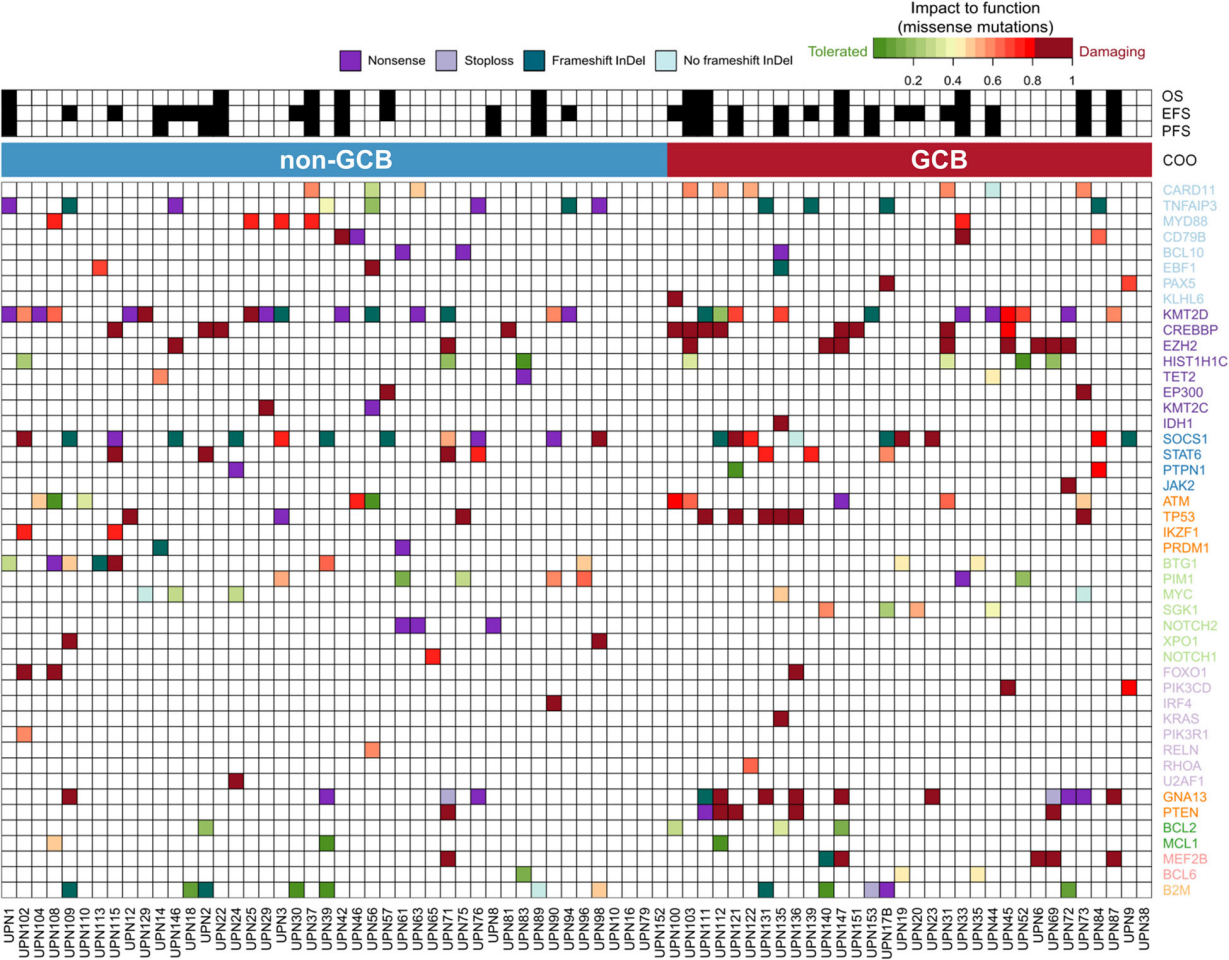
Overview of detected mutations and clinical endpoints. In the *upper part* of the figure, the occurrence of an event for each clinical endpoint is indicated by a *filled-in square*. In the *lower part*, mutations are color-coded by their type. Additionally, missense mutations are color-coded according to their predicted impact on protein function (MetaLR rank score, see the “Methods” section) Genes (*in rows*) are grouped according to their involvement in the cellular pathway and ordered according to their mutation frequencies within each group. Cases are ordered according to the cell-of-origin subtype. If multiple mutations occurred within one gene in the same case, the most damaging mutation is shown. *COO* cell-of-origin, *EFS* event-free survival, *GCB* germinal center B cell type, *OS* overall survival, *PFS* progression-free survival

We used the ensemble MetaLR score that integrates nine different functional prediction algorithms and population allele frequencies to predict the functional impact of the detected missense mutations. Ninety-eight percent of all missense mutations were successfully annotated, and 86/227 (38%) were reliably predicted to be deleterious. Functional effects did not distribute evenly among investigated genes. In some (*GNA13*, *TP53*, *CREBBP*, *TNFAIP*), all or nearly all substitutions were either nonsense or predicted damaging missense mutations, whereas in others (*PIM1*, *BCL2*), the majority of mutations were “neutral,” and thus likely played a passenger role in lymphomagenesis (Fig. 2).

### Survival analysis

We performed Kaplan–Meier survival analysis of all genes that were mutated in at least 4 cases within our cohort and, also, on combinations of mutated genes that act on the same pathway. Only significant findings are reported.

**Fig. 3:**
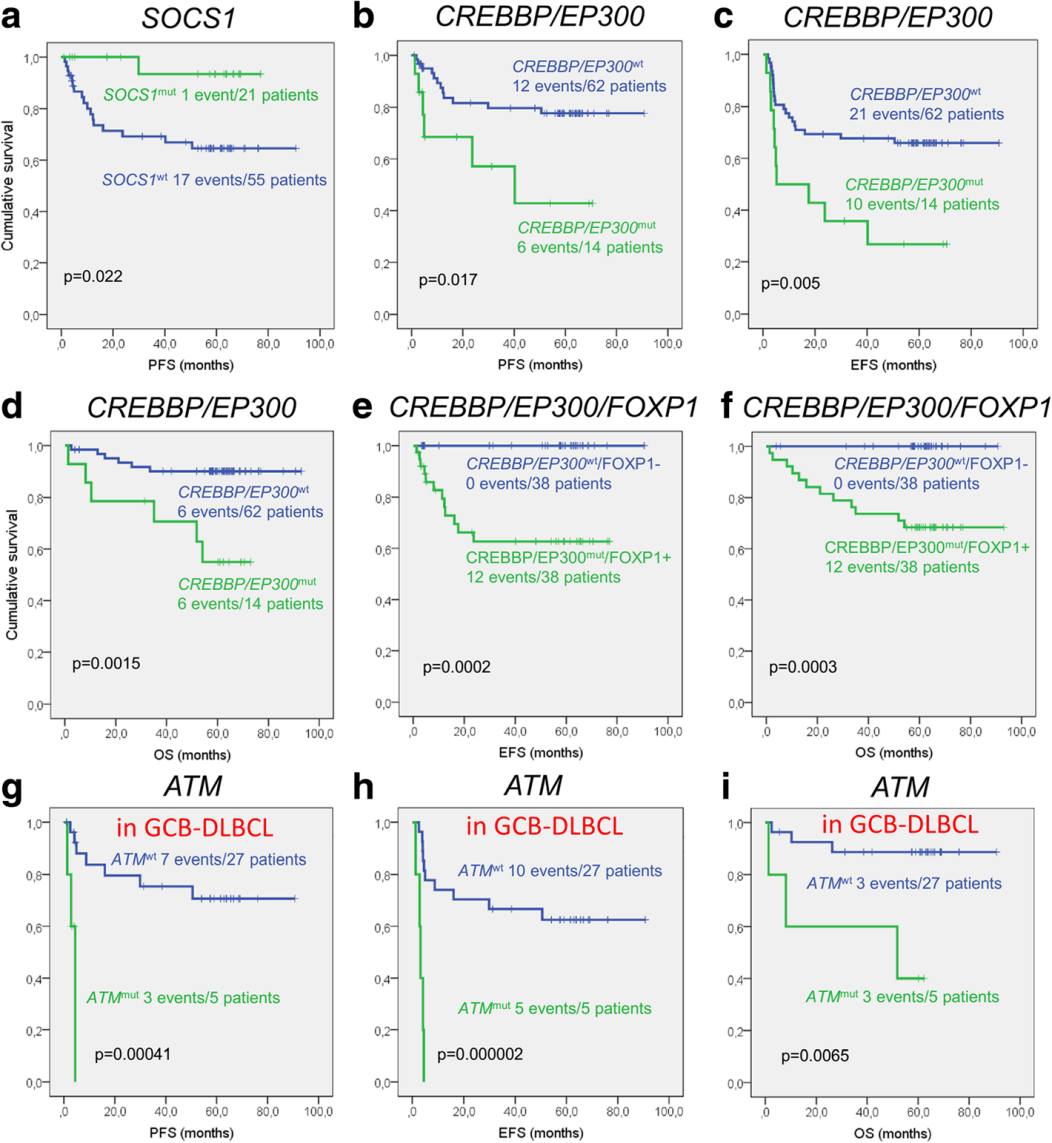
Survival analysis according to mutational status. **a** Progression-free survival (PFS) in *SOCS1*-mutated and non-mutated cases. **b**–**d** Comparison of overall survival (OS), PFS, and event-free survival (EFS) in *CREBBP*- or *EP300*-mutated and wild-type cases. **e**–**f** Separation between cases with good and bad prognosis was further increased by combining *CREBBP* or *EP300* mutational status and FOXP1 protein overexpression. **g**–**i** Prognostic significance of *ATM* mutations in GCB–DLBCL

*SOCS1* mutated cases had better PFS compared to wild-type cases (*p* = 0.022), but the difference was not significant for OS and EFS (*p* = 0.11 and *p* = 0.182, respectively) (Fig. 3a). In total, 47 mutations of *SOCS1* were detected, affecting 21 cases (12 non-GCB and 9 GCB). Mutations were distributed evenly along the coding sequence with two hotspots at Ala17 and Phe79 (four and three mutations, respectively) (Additional file 2: Figure S1). In all but 2 cases, at least one truncating (frameshift insertion/deletion or nonsense) or predicted damaging missense mutation was present, suggesting a deleterious effect on protein synthesis and/or function. Nineteen of 47 (40%) *SOCS1* mutations were potentially induced by aberrant somatic hypermutation (aSHM), as they occurred within the RGYW/WRCY DNA sequence motif known to be targeted by activation-induced deaminase.

Since *CREBBP* and *EP300* have close functional interactions in acetylating histone and nonhistone proteins, we decided to evaluate their combined prognostic value. Cases with mutations in *CREBBP* or *EP300* had significantly worse OS, EFS, and PFS (*p* = 0.0015, *p* = 0.005, and *p* = 0.017, respectively) (Fig. 3b–d). In total, 14 cases were affected, with one heterozygous mutation in each case. All detected variants were missense substitutions localized in the acetyltransferase HAT domain with predicted deleterious effects on protein function (Additional file 2: Figure S1). However, the existence of more mutations cannot be excluded, since the applied gene panel only partly investigated the coding sequence of both *CREBBP* and *EP300. CREBBP* mutations alone had a negative impact on OS, but this did not reach statistical significance (*p* = 0.061). Analysis of potential linkage of these mutations to aSHM showed no such evidence.

In our previous study, we reported that overexpression of FOXP1 evaluated by IHC was prognostic of worse OS [13]. We combined mutation- and IHC-based prognostic markers to determine whether this improved identification of DLBCL cases with poor outcomes. Fifty-one percent (39/76) of cases overexpressed FOXP1 and/or had mutations of *CREBBP/EP300*. Five tumors shared both of these features. Survival analysis showed significantly worse OS, PFS, and EFS in cases with either FOXP1 overexpression or *CREBBP or EP300* mutations (*p* = 0.0003, *p* = 0.006, and *p* = 0.0002, respectively) (Fig. 3e–f).

*ATM* mutations emerged as prognostic factor for worse EFS, PFS, and OS within the GCB–DLBCL subgroup (*p* = 0.000002, *p* = 0.00041, and *p* = 0.0065, respectively; Fig. 3g–i). In total, 11 mutations affecting 10 DLBCL cases were found (5 in GCB and 5 in non-GCB instances). Only in one case *ATM* mutations were reliably predicted to be deleterious, while the functional significance of other mutations remained unclear. Also, there was no evidence of targeting of this gene by aberrant somatic hypermutation as only 2/11 mutations occurred within the RGYW motif.

In multivariable analysis that included IPI and failure to achieve complete remission (FACR), *CREBBP* and *EP300* mutational status (but not *SOCS1* status) emerged as the most significant factor predicting worse OS (*p* = 0.013, HR 4.12, CI 1.38–14.15) (Table 3). While these mutations were also significant for predicting worse EFS and PFS (*p* = 0.021 and *p* = 0.041, respectively), FACR was a superior predictor of the latter two endpoints (*p* = 0.0006 and *p* = 0.001).

**Table 3:** Stepwise Cox regression analysis of survival

	OS		EFS		PFS	
Variable	*p* value	HR (95% CI)	*p* value	HR (95% CI)	*p* value	HR (95% CI)
*CREBBP* ^mut^ or *EP300* ^mut^	0.013	4.41 (1.38–14.16)	0.021	2.53 (1.15–5.54)	0.041	2.94 (1.05–8.27)
Failure to achieve complete remission	0.032	0.27 (0.08–0.89)	0.0006	0.22 (0.097–0.53)	0.001	0.17 (0.056–0.49)
International Prognostic Index (IPI)	0.86	0.96 (0.61–1.51)	0.38	0.88 (0.66–1.17)	0.646	1.09 (0.75–1.60)

## Discussion

There is a critical need for additional tumor-based prognostic markers in DLBCL to identify high-risk DLBCL patients prior to first treatment that might benefit from alternative risk-adjusted therapies. Tumor mutations represent promising candidates for outcome prognostication due to the unequivocal nature of results obtained by respective mutational detection techniques and the increased availability and robustness of HTS. We have demonstrated that targeted mutational analysis of a relatively small but uniformly treated and prospectively followed up patient cohort can reveal significant prognostic associations in DLBCL. This was only possible (a lesson learned) because of the proper trial design contemplating central collection of tissue for translational analysis as an integral study part. Additional lessons learned from this prospective trial such as the importance of a central diagnostic pathology review as well as handling of biological entities and subentities in the spectrum of so-called high grade B cell lymphomas have been discussed in a paper of ours recently published in this journal [13].

We found that deleterious mutations in two acetyltransferase genes, *CREBBP* and *EP300*, which belong to the KAT3 family of histone/protein lysine acetyltransferases, predict worse OS, PFS, and EFS in DLBCL independent of IPI and FACR. Point mutations or deletions of *CREBBP/EP300* reportedly affect 39% of all DLBCL cases [31]. In line with other studies, we confirm that *CREBBP* mutations are more frequent than *EP300* and tend to occur more often in GCB–DLBCL [18]. In the context of cancerogenesis, *CREBBP* and *EP300* act as tumor suppressors. It has been shown that loss of one *CREBBP* allele leads to reduced acetylation and inactivation of p53, impaired expression of glucocorticoid-receptor-responsive genes, and upregulation of BCL6 [31, 32]. Finally, recent data suggest that heterozygous deleterious *CREBBP* mutations lead to decreased global histone H3 lysine 14 (H3K14), K18, and K27 acetylation and reduced MHC class II expression (Hashwah et al., currently under peer review). Association with poorer outcomes were suggested in studies that found *CREBBP* mutations in 20% of relapsed/refractory GCB–DLBCL [33], and in a large proportion of relapsed acute lymphoblastic leukemia patients [31, 34]. Despite this important clue, however, the prognostic value of *CREBBP/EP300* mutations in DLBCL has not been previously reported.

*SOCS1* mutations predicted excellent PFS in our cohort, but differences in OS and EFS were not significant. Suppressor of cytokine signaling 1 (*SOCS1* ) is a known inhibitor of JAK/STAT-dependent signal transduction, which binds to phosphorylated JAK and marks it for proteosomal degradation [35, 36]. Mutations of *SOCS1* have been previously shown to be associated with favorable survival in DLBCL [37] and are also frequently detected in Hodgkin lymphoma and primary mediastinal B cell lymphoma (PMBCL), both with a relatively favorable prognosis [38]. We also previously showed that *SOCS1* mutations occurred exceptionally in non-relapsing primary DLBCL whereas being completely absent in relapsing DLBCL cases, supporting the association with favorable prognosis [27]. Schif et al. reported that DLBCL bearing truncating *SOCS1* mutations have excellent OS, whereas those with only missense mutations have markedly worse prognosis [37]. Our data, however, does not confirm such distinction: 6 of 19 *SOCS1*-mutated cases had only missense mutations, but their PFS was equally favorable as those with truncating mutations. The *SOCS1* mutational frequency in our cohort (28%) was higher than reported average (~13%) [39]. All detected mutations had relatively high variant allelic frequencies (median 28%, range 6–78%); therefore, our higher mutation rate cannot be explained by comparably high sequencing depth and sensitive detection of subclonal mutations that could have been missed by exome-scale sequencing studies. A potential explanation might be bias of our cohort toward better than average survival, e.g., due to study protocol exclusion of patients with performance status >2 on the ECOG scale, with symptomatic central nervous system disease, or with HIV, and/or hepatitis infection, thus being potentially enriched for cases with better prognosis that consequently more often bear *SOCS1* mutations.

The prognostic value of multiple other gene mutations such as *TP53*, *MYD88*, *FOXP1*, and *FOXP2* in DLBCL has been suggested previously [22, 23, 40, 41]. In our cohort, *TP53*-mutated cases had worse OS, but this difference did not reach statistical significance. Also, the low number (*n* = 5) of *MYD88* L265P-mutated cases did not allow detection of any reliable prognostic associations. Analogously, despite *ATM* mutations were consistently associated with worse outcomes in GCB–DLBCL in our study, this observation was based on a small number of events and cases bearing mutations and therefore remains to be validated on other larger collectives. While we were unable to identify mutations of *FOXP1*, we previously reported that overexpression of the FOXP1 protein is associated with worse OS in the investigated DLBCL cohort [13]. Here, we show that combination of HTS- and IHC-based prognostic markers (*CREBBP/EP300* mutations and FOXP1 overexpression) enables even better stratification between patients with good and worse prognosis.

It is unlikely that any single biomarker will significantly improve risk stratification in DLBCL due to the profound heterogeneity of this disease. This point is illustrated by 3 cases in our cohort with mutations of both *SOCS1* and *CREBBP* (Fig. 2, cases UPN57, UPN112, and UPN115) that clinically behaved like *CREBBP-*mutated cases and had worse prognosis. It is more likely that a combination of several different types of markers established on uniformly treated prospective cohorts and selected by robust statistical methods would provide models that could be prospectively validated on larger DLBCL collections. A good example of such an updated composite prognostic model is the m7-FLIPI, a recently developed score for follicular lymphoma, which incorporates mutations and clinical factors and provides superior prognostication compared to traditionally used clinical factor-based FLIPI [42].

## Conclusion

Deleterious mutations in the HAT domain of the acetyltransferases *CREBBP* and *EP300* are associated with worse OS, PFS, and EFS in DLBCL. *ATM* mutations are prognostic for worse survival at all clinical endpoints in GCB–DLBCL, but due to low case number remains to be verified on larger collectives. The previously reported beneficial prognostic role of *SOCS1* mutations in DLBCL is valid for predicting better PFS.

## Additional files

Additional file 1: Table S1. Statistics of high-throughput sequencing and variant calling. **Table S2.** Somatic mutations detected by high-throughput sequencing in 76 cases of primary DLBCL. (XLSX 384 kb)
Additional file 2: Figure S1. Mapping of the detected mutations to the protein sequences within respective domains in the ten most frequently mutated and relevant genes is this study. Mutations in *CREBBP* and *EP300* always occurred within the HAT domain responsible for acetylation. In *SOCS1*, mutations were distributed evenly along the protein sequence. *Y*-axis represents the number of mutations detected at a particular amino acid position. Green–missense mutation; black–frameshift insertion/deletion; purple–nonsense mutation. (PDF 442 kb)

## Abbreviations

ASCT: Autologous stem cell transplantation
aSHM: Aberrant somatic hypermutation
BAM: Binary sequence alignment/map format
CI: Confidence interval
COO: Cell-of-origin
DLBCL: Diffuse large B cell lymphoma
EBER: Epstein-Barr virus early RNA
ECOG: Eastern Cooperative Oncology Group
EFS: Event-free survival
FACR: Failure to achieve complete remission
FFPE: Formalin-fixed paraffin-embedded
FISH: Fluorescence in situ hybridization
FLIPI: Follicular Lymphoma International Prognostic Index
GCB: Germinal center B cell
HTS: High-throughput sequencing
IHC: Immunohistochemistry
IPI: International Prognostic Index
OS: Overall survival
PET/CT: Positron emission tomography/computed tomography
PMBCL: Primary mediastinal B cell lymphoma
PFS: Progression-free survival
R-CHOP: Rituximab, cyclophosphamide, hydroxydaunorubicin, vincristine, and prednisone
