# Supplementary material for: Mutations of CREBBP and SOCS1 are independent prognostic factors in diffuse large B cell lymphoma: mutational analysis of the SAKK 38/07 prospective clinical trial cohort
Source: J Hematol Oncol. 2017 Mar 17;10:70. doi: 10.1186/s13045-017-0438-7 (PMC5356266; doi:10.1186/s13045-017-0438-7)
Supplement: Additional file 2: Figure S1. — Mapping of the detected mutations to the protein sequences within respective domains in the ten most frequently mutated and relevant genes is this study. Mutations in CREBBP and EP300 always occurred within the HAT domain responsible for acetylation. In SOCS1, mutations were distributed evenly along the protein sequence. Y-axis represents the number of mutations detected at a particular amino acid position. Green–missense mutation; black–frameshift insertion/deletion; purple–nonsense mutation. (PDF 442 kb) [file 13045_2017_438_MOESM2_ESM.pdf]

ATM

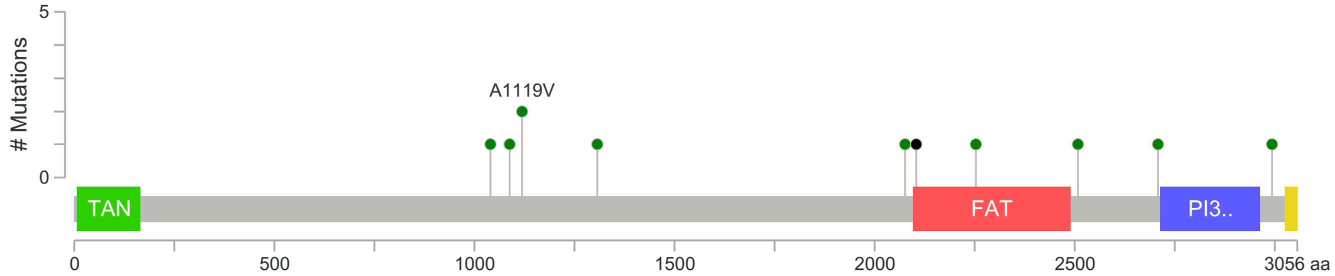

B2M

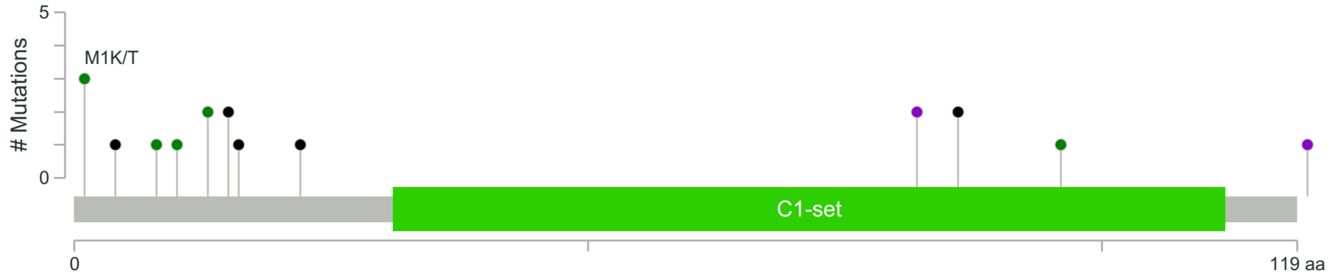

BTG1

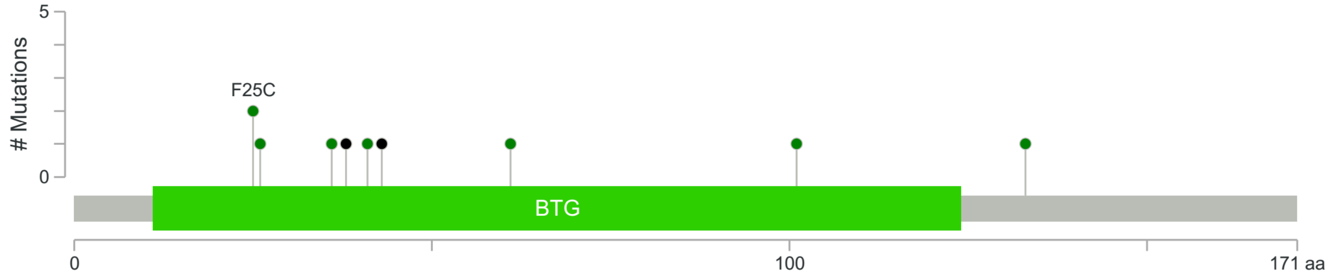

CARD11

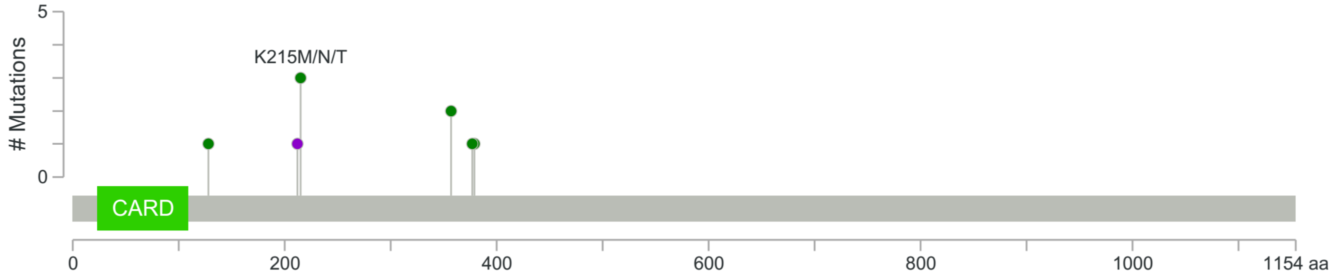

CREBBP

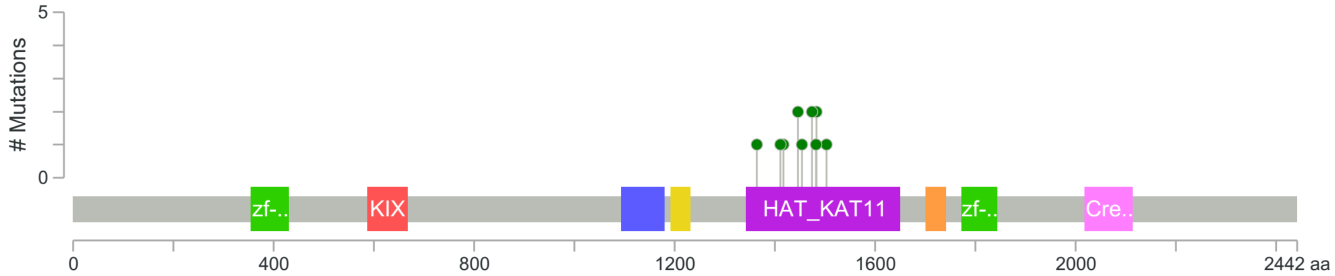

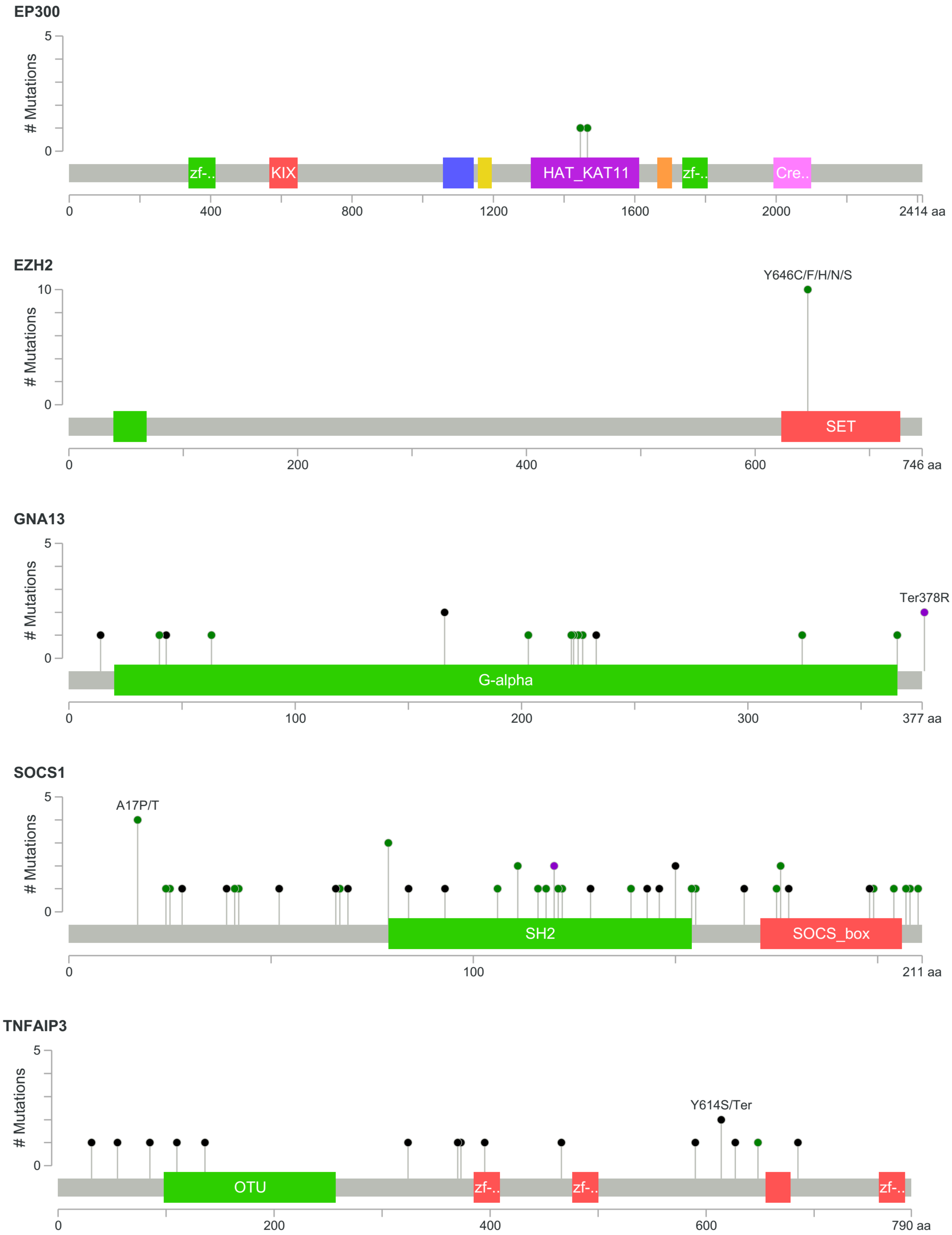

Supplementary Figure 1. Mapping of the detected mutations to the protein sequences within respective domains in the 10 most frequently mutated and relevant genes is this study. Mutations in *CREBBP* and *EP300* always occurred within the HAT domain responsible for acetylation. In *SOCS1*, mutations were distributed evenly along the protein sequence. Y-axis represents number of mutations detected at a particular amino acid position. green – missense mutation; black – frameshift insertion/deletion; purple – nonsense mutation.
